# Cumulative effects of widespread landscape change alter predator–prey dynamics

**DOI:** 10.1038/s41598-022-15001-3

**Published:** 2022-07-08

**Authors:** Nicole P. Boucher, Morgan Anderson, Andrew Ladle, Chris Procter, Shelley Marshall, Gerald Kuzyk, Brian M. Starzomski, Jason T. Fisher

**Affiliations:** 1 School of Environmental Studies, University of Victoria, Victoria, BC V8W 2Y2 Canada; 2 British Columbia Ministry of Forests, 2000 South Ospika Boulevard, Prince George, BC V2N 4W5 Canada; 3 British Columbia Ministry of Forests, 1259 Dalhousie Drive, Kamloops, BC V2C 5Z5 Canada; 4 British Columbia Ministry of Forests, 2080 Labieux Road, Nanaimo, BC V9T 6J9 Canada; 5 Government of Saskatchewan Fish, Wildlife and Lands Branch, Ministry of Environment, Unit #1-101 Railway Place, Box 607, Meadow Lake, SK S9X 1Y5 Canada

**Keywords:** Ecology, Behavioural ecology, Ecosystem ecology

## Abstract

Predator search efficiency can be enhanced by anthropogenic landscape change, leading to increased predator–prey encounters and subsequent prey population declines. Logging increases early successional vegetation, providing ungulate forage. This increased forage, however, is accompanied by linear feature networks that increase predator hunting efficiency by facilitating predator movement and increasing prey vulnerability. We used integrated step selection analyses to weigh support for multiple hypotheses representing the combined impact of logging features (cutblocks and linear features) on wolf (*Canis lupus*) movement and habitat selection in interior British Columbia. Further, we examine the relationship between logging and wolf kill-sites of moose (*Alces alces*) identified using spatiotemporal wolf location cluster analysis. Wolves selected for linear features, which increased their movement rates. New (0–8 years since harvest) cutblocks were selected by wolves. Moose kill-sites had a higher probability of occurring in areas with higher proportions of new and regenerating (9–24 years since harvest) cutblocks. The combined selection and movement responses by wolves to logging features, coupled with increased moose mortality sites associated with cutblocks, indicate that landscape change increases risk for moose. Cumulative effects of landscape change contribute to moose population declines, stressing the importance of cohesive management and restoration of anthropogenic features.

## Introduction

Anthropogenic landscape change modifies predator–prey dynamics, which has implications for both predator and prey populations^1,2^. Predators respond to prey through numerical responses (changes in predator density) and functional responses (changes in consumption rate), and mathematical models describing the functional response identify limits to the rate of prey consumption, prey encounter rate, and handling time^3,4^. Holling’s disc equation, which models the functional response, postulates that kill rate is limited by handling time at high prey densities, but by search efficiency at low prey densities^3,4^. Growing evidence indicates that anthropogenic landscape change influences predator search efficiency and thus, predator–prey encounter rate by facilitating predator movement and/or altering prey vulnerability^1,5,6^. Unless prey alter their behavior to avoid predation (e.g. sheltering in human-created refugia^7^) or landscape change bolsters prey populations by increasing habitat quality (e.g., increasing available forage), anthropogenic landscape change could lead to declining prey populations due to increased predator foraging efficiency.

Predators exploit specific anthropogenic features to increase search efficiency, which intensifies predation risk for prey^8^. Large carnivores often select and travel quickly on linear features, which improves foraging efficiency by increasing potential predator–prey encounters^1,5,9^. Logging—which creates both roads and cutblocks—increases predator travel efficiency, reduces hiding cover, concentrates prey in remaining patches, and creates predictable, small areas for predators to search^10,11^. Additionally, prey species are attracted to polygonal features such as cutblocks, where early seral vegetation offers abundant forage^12–14^. If predators hunt more efficiently due to linear features linking cutblocks^5,15^, these anthropogenic features could function cumulatively to increase predation risk for prey across disturbed landscapes.

Predation risk could be elevated if prey select for cutblocks (e.g. for increased forage) but logging features also increase predator search efficiency, possibly leading to an ecological trap^16–18^. In areas with extensive logging, such as forests infested with outbreaks of bark beetles (Scolytinae) which are subsequently logged to salvage timber^19,20^, the opportunity for such scenarios to manifest may be intensified^16–18^. Landscapes highly modified by salvage logging of beetle-killed forests are characterized by cutblocks that are significantly larger than conventional cutblocks, linked by extensive linear feature networks and interspersed with patches of forests relatively homogeneous in structure, age, and composition^21,22^. If functioning cumulatively to increase predation risk, salvage logging features could lead to prey population declines. This may be the case in the western sub-boreal, where extensive salvage logging of forest killed by mountain pine beetle (MPB; *Dendroctonus ponderosae*) outbreaks linked to climate change coincided with declines in moose (*Alces alces*) populations^20,23–25^.

One mechanism hypothesised for the moose population decline within interior BC could be increased movement rates of wolves (*Canis lupus*)—a primary predator of moose—and altered habitat selection, resulting in increased predation risk for moose near logging features^25^. Evidence suggests that anthropogenic landscape change—particularly, linear feature networks—facilitates predation by wolves^1,5,26^. Selection for linear features increases wolf movement efficiency, affecting predator–prey encounter rates and subsequent predation rates on ungulates^1,5,9,27–30^. Additionally, wolves select forest edges, cutblocks, and areas with new forage created by logging, due to increased availability of prey associated with these features^14,15,31,32^. We argue that anthropogenic features facilitating wolf travel and creating predictable prey locations is a concern for moose inhabiting areas undergoing extensive logging.

We examined whether the two dominant forms of anthropogenic landscape change associated with salvage logging—linear features and cutblocks—work to cumulatively influence wolf movement and habitat selection, and are tied to moose kill-site locations within our study area—interior British Columbia (BC), Canada. We ask: (1) do wolves select for salvage logging features; (2) do salvage logging features facilitate wolf movement; and (3) are salvage logging features linked to wolf kill-sites of moose? We hypothesized that the impacts of cutblocks and linear features function together to affect predator–prey dynamics. Specifically, we predict that wolves will select for cutblocks and linear features and have increased movement rates associated with these disturbance features. We expect that cutblock age and size will influence wolf habitat selection, with increased selection for smaller, regenerating (9–24 years since harvest) cutblocks due to increased prey availability^14,33,34^. Lastly, we expect these landscape features to facilitate wolf predation on moose, such that there is a positive relationship between salvage logging features and wolves’ kill-sites of moose.

## Methods

### Study area

BC’s Interior Plateau has experienced the cumulative effects of significant land conversion and habitat loss, with impacts to forests including a recent severe MPB outbreak^35–37^. This outbreak began in the 1990s, killing over 53% of merchantable pine (723 million m^3^ of pine)^35,36,38^. To mitigate economic effects, the Government of BC increased the annual allowable cut (timber amount sustainably harvested per year for a region) by approximately 30% from levels prior to the outbreak, resulting in extensive linear feature networks and the removal of large areas of beetle-killed trees^36^.

**Figure 1:**
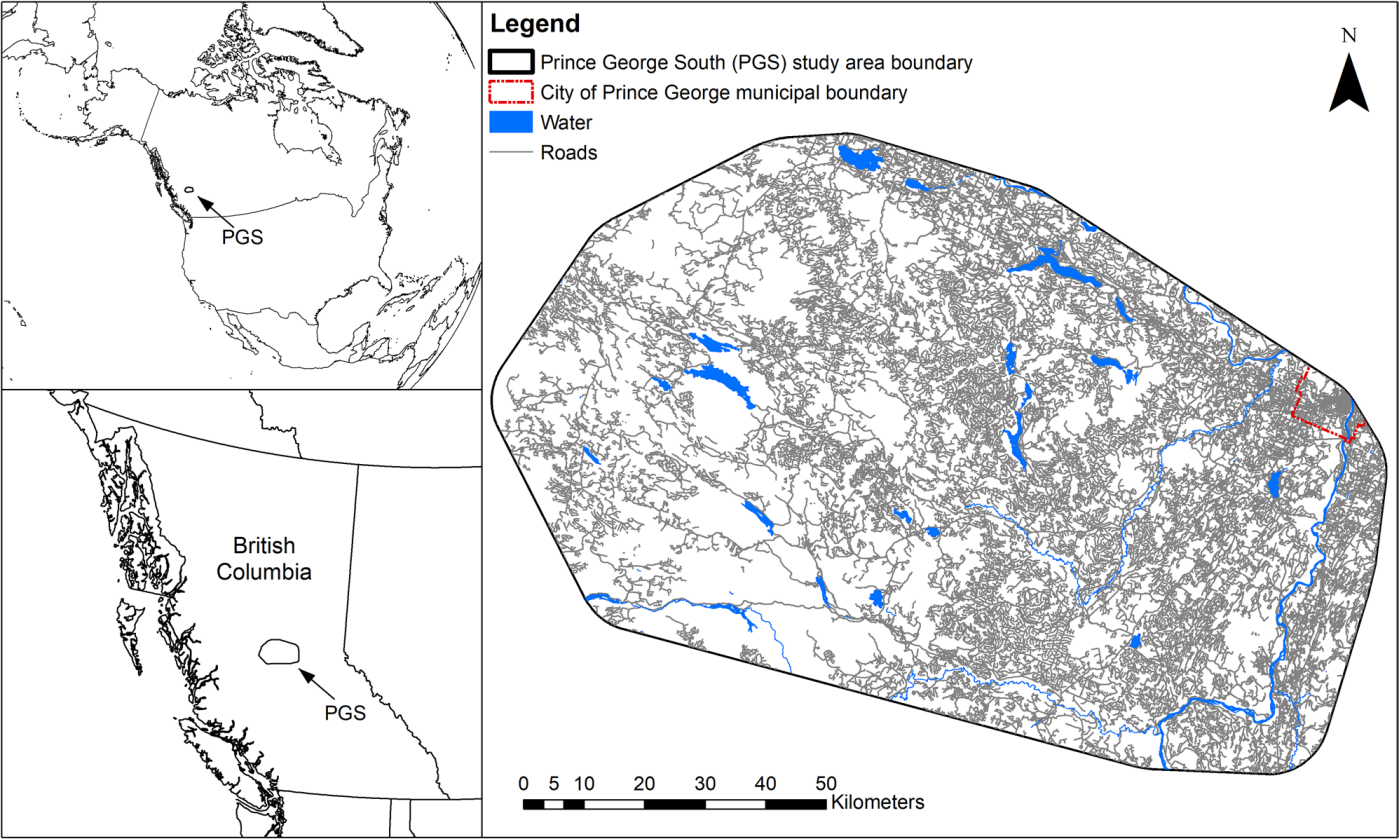
The Prince George South (PGS) study area, located in interior British Columbia, Canada. PGS is heavily altered with linear features (in grey) and cleared forest. The municipal boundary for the city of Prince George is outlined in red. Waterbodies are shown in blue. The map was created in ArcGIS v.10 (www.esri.com).

Our study area, Prince George South (PGS), is located southwest of the city of Prince George, on BC’s Interior Plateau (Fig. 1; Supplementary Information S1). PGS is one of five study areas in a long-term provincial moose monitoring project, selected for additional research on predation dynamics due to its continued moose declines and the identified role of wolves as a leading cause of mortality for both adult females and 8–12 month old calves^25^.

### Integrated step selection analysis

Adult wolves were captured using standard protocols for aerial darting or net-gunning (December-March), or soft-catch, foot-hold trapping (June–July), 2018–2020 (Supplementary Information S1). All wolf captures and protocols were completed following Canadian Council on Animal Care and Animal Research: Reporting In Vivo Experiments (ARRIVE) guidelines, as well as BC Ministry of Environment Standards for Live Animal Capture and Handling. Approval for experimental protocols and animal care guidelines was issued in accordance with the British Columbia Wildlife Act and BC Ministry of Forest, Lands and Natural Resource Operations Animal Care Ethics Committee (permit: PG17-272811).

Captured wolves were fitted with satellite GPS collars (Vectronic Aerospace, Berlin) with a 60-min fix rate and 2-year drop-off mechanisms. Wolf movement data was divided into ‘winter’ (October 1-March 31; snow present, no pups) and ‘summer’ (April 1-September 30; denning, rearing pups, ungulate calving, snow-free) periods. We were interested in wolf habitat selection and movement during all periods except those associated with denning and rendezvous sites, where we assumed there would be limited prey searching behaviors by wolves^39^. We removed wolf locations within 1 km of these sites^40^, determined using GPS cluster analysis^41^ and ground truthing. We removed GPS locations within the first 48-h after capture to account for altered behavior following handling and only included wolves with > 7 days of movement data.

Integrated step selection analyses (iSSA) compare used (1) to available (0) locations of steps (connection between successive relocations), integrating habitat selection and movement within a conditional logistic regression model framework^42^. We used the R package ‘amt’ (Animal Movement Tools Version 0.0.6.) to generate ten random steps for every used wolf step^43,44^, drawn from population-level parametric distributions of step lengths (Euclidean distance between successive relocations) and turn angles (angle between consecutive relocations). Because we had a limited sample size of wolves and packs, we used individual wolves as the sampling unit and retained all individuals regardless of pack within the analysis. While this decision could lead to biased results due to pseudo-replication and territory restrictions, there is evidence suggesting that individual resource use varies between pack members^45^.

We included the following habitat covariates: cutblock use (new cutblock [0–8 years since harvest], regenerating cutblock [9–24 years since harvest] or outside of cutblock [reference category]^33^) and size; distance to, and density of, linear features; distance to edge habitat; land cover type (deciduous-leading stands, coniferous-leading stands, mixed forest stands, pine*-*leading stands, and non-forest); plant productivity (normalized difference vegetation index, NDVI); and distance to the nearest waterbody (Supplementary Information S1). All environmental covariates that were included as an interaction with ln(Step length) were extracted from the start of the step, while all other covariates were extracted from the end of the step. Distance covariates were log-transformed to account for skewness.

We developed candidate models with each model representing a competing hypothesis (Table 1), and modeled iSSAs for each individual in each season separately^42,46^. As step length may vary with time of day, we created a harmonic interaction term, hereafter referred to as sin(hour), to represent activity peaks at dawn and dusk, using the following formula: sin($\frac{4*\pi *(hour-6)}{24}$)^47,48^. All models included ln(Step length) and an interaction between sin(hour) and ln(Step length) to control for varying movement rates at different times of day (Supplementary Fig. S1).

**Table 1:** Candidate models for the integrated step selection analysis examining wolf (*Canis lupus*) movement and habitat selection in Prince George South, 2018–2020.

Model name	Covariates
Prey	ln(SL) + ln(SL):sin(hour) + Pine + Deciduous + Mixed Forest + Coniferous + ln(Distance to water) + NDVI + ln(Edge in) + ln(Edge out)
LF network	ln(SL) + ln(SL):sin(hour) + LF density + ln(Distance to LF) + ln(SL):ln(Distance to LF) + ln(SL):LF density
Cutblock	ln(SL) + ln(SL):sin(hour) + NC + RC + NC:Cut size + RC:Cut size + ln(SL):NC + ln(SL):RC + ln(SL):NC:Cut size + ln(SL):RC:Cut size
Prey + LF network	ln(SL) + ln(SL):sin(hour) + Pine + Deciduous + Mixed Forest + Coniferous + ln(Distance to water) + NDVI + ln(Edge in) + ln(Edge out) + LF density + ln(Distance to LF) + ln(SL):ln(Distance to LF) + ln(SL):LF density
Prey + Cutblock	ln(SL) + ln(SL):sin(hour) + Pine + Deciduous + Mixed Forest + Coniferous + ln(Distance to water) + NDVI + ln(Edge in) + ln(Edge out) + NC + RC + NC:Cut size + RC:Cut size + ln(SL):NC + ln(SL):RC + ln(SL):NC:Cut size + ln(SL):RC:Cut size
LFN + Cutblock	ln(SL) + ln(SL):sin(hour) + LF density + ln(Distance to LF) + ln(SL):ln(Distance to LF) + ln(SL):LF density + NC + RC + NC:Cut size + RC:Cut size + ln(SL):NC + ln(SL):RC + ln(SL):NC:Cut size + ln(SL):RC:Cut size
Global	ln(SL) + ln(SL):sin(hour) + Pine + Deciduous + Mixed Forest + Coniferous + ln(Distance to water) + NDVI + ln(Edge in) + ln(Edge out) + LF density + ln(Distance to LF) + ln(SL):ln(Distance to LF) + ln(SL):LF density + NC + RC + NC:Cut size + RC:Cut size + ln(SL):NC + ln(SL):RC + ln(SL):NC:Cut size + ln(SL):RC:Cut size

ln() = log-transformed covariate.

*SL* step length, *LF* linear feature, *NC* new cut, *RC* regenerating cut.

Akaike’s Information Criterion (AIC) was used to determine the best-supported model for each individual wolf in each season. Performance of models was assessed using cross-validation, with data subset by step ID. For model selection, we determined the best overall model for each season by assessing the distribution of AIC weights. Then, we used bootstrapping to estimate population β coefficients and associated confidence intervals from the best-supported model^46,47,49,50^. This two-stage approach of fitting separate individual models and then post-hoc estimating population averages via bootstrapping is commonly used for iSSAs when sample sizes for individual steps are sufficient^42^. This approach allows for unbiased estimation of habitat selection variability and fewer assumptions than mixed-effects models^42,46^. For bootstrapping, we weighted samples by individual wolf i.d., which ensured equal probability of sampling for each individual wolf. From 2000 repetitions, we obtained the median and confidence interval for beta coefficient estimates (using 2.5th and 97.5th quantiles) which were used for population-level inferences. To quantify selection responses, we calculated relative strength of selection which estimates probability of selecting one resource unit over another^51^ (Supplementary Information S1).

### Moose kill-site analysis

Moose mortality sites were determined by ground-truthing potential kill-sites identified by cluster analysis of wolf GPS locations, using the Find Points Cluster Identification Program Version 2^41^ (Supplementary Information S1). We used logistic regressions to compare habitat features at sites of successful wolf kills of moose to random sites selected within the study area. We used variance inflation factors (VIF) to check for multicollinearity and excluded variables with VIF > 4. We weighed evidence for competing hypotheses relating landscape features to sites where moose were killed by wolves, following a set of a priori candidate models similar to the iSSA set (Supplementary Table S2), and selected the best supported model using AIC. For the top model, we used k-fold cross validation with k = 10 and Spearman’s rank correlation (r_s_) to assess model fit^52^.

## Results

We deployed satellite GPS collars on ten wolves in five packs (Supplementary Table S1) and collected hourly location data between February 24, 2018 and July 31, 2020. Wolf collars were staggered in deployment and end date, so data was not available from all individuals through the study duration (Supplementary Table S1).

**Figure 2:**
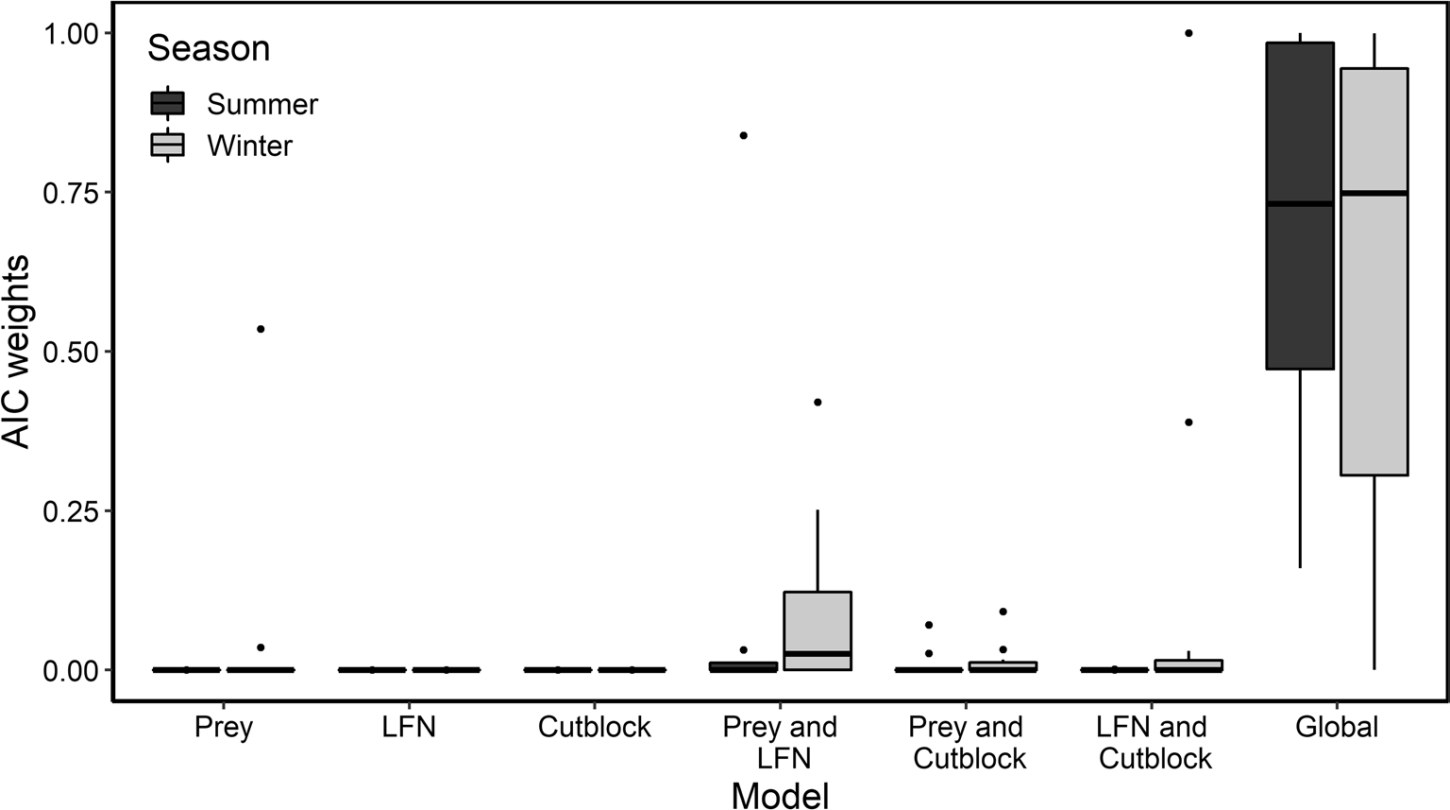
Akaike’s information criterion (AIC) weight distribution for summer (April 1–September 30) and winter (October 1–March 31) integrated step selection analysis candidate models for Prince George South, 2018–2020. Outliers are represented by points.

For both seasons, the ‘Global’ model outperformed the alternate models (Fig. 2, Table S3), indicating wolf movement and habitat selection is influenced by a combination of cutblocks, linear features and natural features. All remaining models received minimal support based on AIC weights.

### Wolf selection for salvage logging features

**Figure 3:**
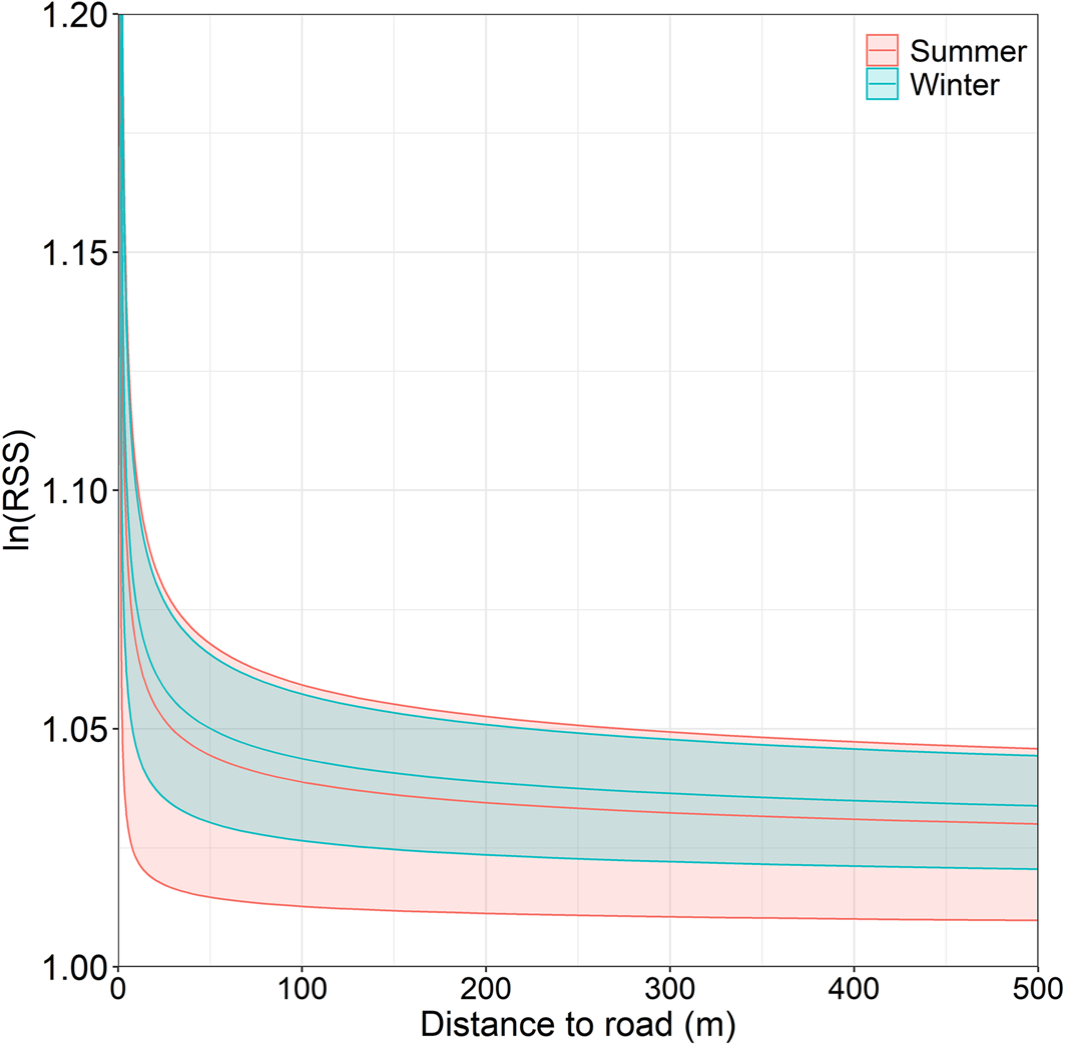
Seasonal wolf log-transformed relative selection strength (RSS) with 95% confidence intervals for distance to linear features (m) in summer (April 1–September 30) and winter (October 1–March 31) for Prince George South, 2018–2020.

In both seasons, wolves selected habitat closer to linear features (Table 2, Fig. 3). There was no clear trend in selection of varying linear feature densities for both seasons (Table 2).

**Table 2:** Seasonal habitat selection and movement beta coefficient estimates with lower and upper 95% confidence bounds for the global integrated step selection analysis model in summer (April 1–September 30) and winter (October 1–March 31) in Prince George South, 2018–2020.

Season	Covariate	Lower	Median	Upper
Summer	**Coniferous**	0.074	0.152	0.255
	Deciduous	− 0.059	0.145	0.297
	**ln(Edge in)**	− 0.146	− 0.127	− 0.114
	**ln(Edge out)**	− 0.152	− 0.146	− 0.125
	**ln(LF distance)**	− 0.106	− 0.08	− 0.071
	**ln(Water)**	− 0.081	− 0.069	− 0.021
	Mixed forest	− 0.036	0.131	0.279
	NDVI	− 0.233	1.217	1.475
	**New cut**	0.033	0.183	0.675
	**New cut:Cut size**	− 0.288	− 0.174	− 0.052
	Pine	− 0.042	0.081	0.486
	Regenerating cut	− 0.334	− 0.211	0.285
	Regenerating cut:Cut size	− 0.226	− 0.176	0.036
	LF density	− 0.159	− 0.086	0.034
	**ln(SL)**	0.325	0.381	0.435
	ln(SL):New cut	− 0.109	− 0.046	0.007
	ln(SL):New cut:Cut size	− 0.008	0.054	0.216
	**ln(SL):Regenerating cut**	− 0.063	− 0.044	− 0.004
	ln(SL):Regenerating cut:Cut size	− 0.012	0.009	0.051
	**ln(SL):LF density**	− 0.05	− 0.033	− 0.031
	**ln(SL):ln(Distance to LF)**	− 0.062	− 0.051	− 0.049
	ln(SL):sin(hour)	− 0.002	0.067	0.081
Winter	Coniferous	− 0.212	− 0.14	0.497
	Deciduous	− 0.061	0.018	0.133
	**ln(Edge in)**	− 0.106	− 0.064	− 0.026
	**ln(Edge out)**	− 0.172	− 0.074	− 0.042
	**ln(LF distance)**	− 0.135	− 0.122	− 0.073
	**ln(Water)**	− 0.092	− 0.057	− 0.022
	Mixed forest	− 0.113	− 0.089	0.061
	**NDVI**	0.272	0.365	1.553
	**New cut**	0.031	0.097	0.14
	New cut:Cut size	− 0.135	0.051	0.091
	Pine	− 0.285	− 0.079	0.147
	Regenerating cut	− 0.131	− 0.031	0.052
	Regenerating cut:Cut size	− 0.204	− 0.08	0.042
	LF density	− 0.063	− 0.027	0.009
	**ln(SL)**	0.203	0.249	0.261
	ln(SL):New cut	− 0.113	− 0.04	0.024
	ln(SL):New cut:Cut size	− 0.001	0.023	0.1
	ln(SL):Regenerating cut	− 0.037	− 0.012	0.018
	**ln(SL):Regenerating cut:Cut size**	− 0.013	− 0.01	− 0.003
	**ln(SL):LF density**	− 0.031	− 0.026	− 0.022
	**ln(SL):ln(Distance to LF)**	− 0.041	− 0.036	− 0.028
	ln(SL):sin(hour)	− 0.024	0.019	0.046

‘:’ denotes an interaction between covariates. Bolded terms indicate significance (i.e. beta estimates do not overlap 0). ln() = log-transformed covariate.

*SL* step length, *LF* linear feature.

Wolf selection of logged areas was dependent on cutblock size and age in summer, but only on cutblock age in winter (Table 2). In both seasons, wolves selected for new cutblocks. In summer, selection of new cutblocks decreased as cutblock size increased. There was no clear trend in wolf selection of regenerating cutblocks during both seasons.

In summer, coniferous-leading forests were selected, and in winter, wolves selected for areas with high NDVI values (Table 2). Edge habitats and areas closer to water were selected for in both seasons.

### Impact of salvage logging features on wolf movement

The impact of cutblocks on displacement rates varied between seasons (Table 2). In summer, wolf step lengths were shorter in regenerating cutblocks, but no trend existed in relation to new cutblocks or size of regenerating cutblocks. In winter, displacement rates were only associated with size of new cutblocks, with shorter step lengths as cutblock size increased.

**Figure 4:**
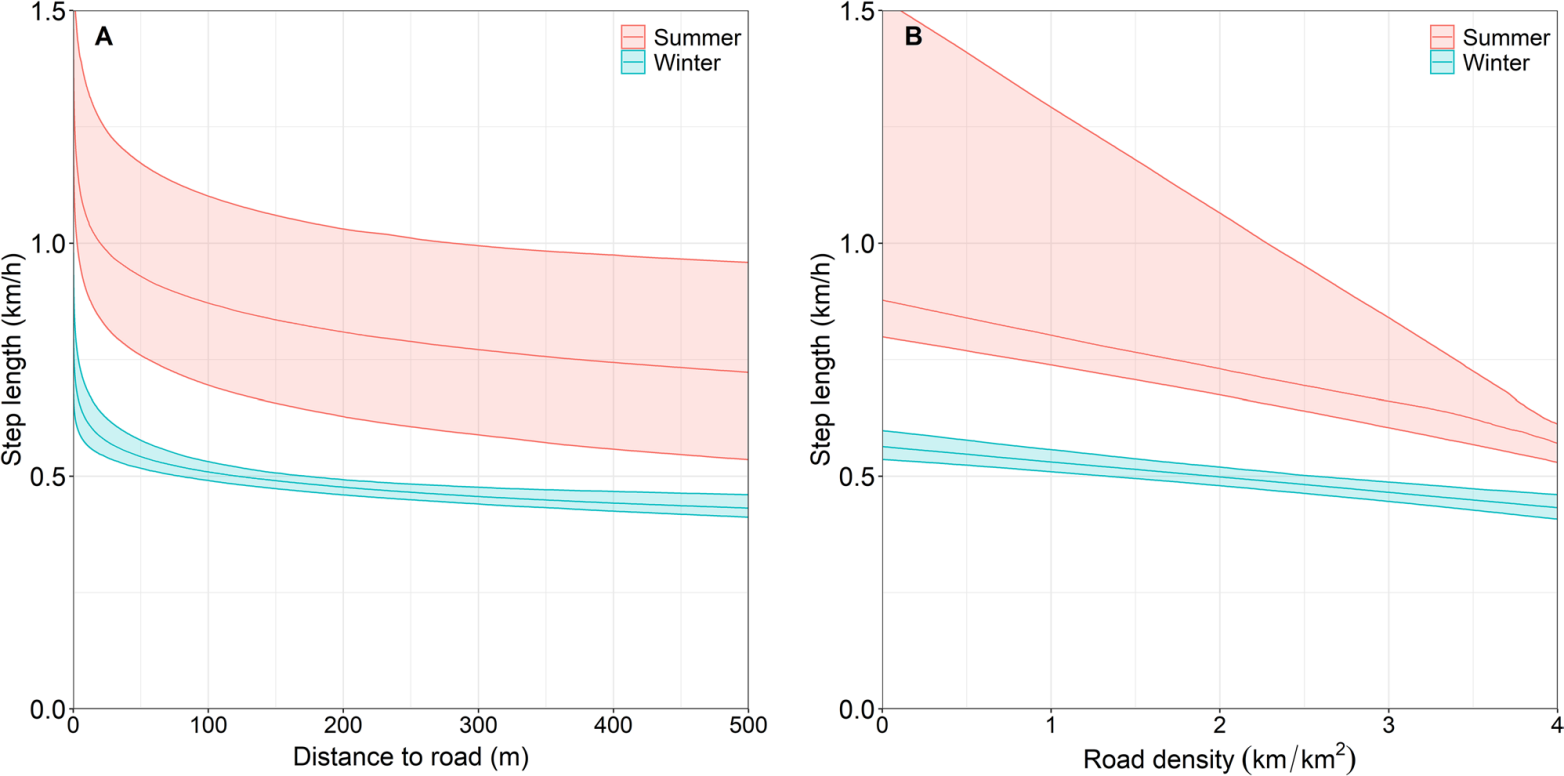
Seasonal mean displacement rates (km/h) with 95% confidence intervals of Prince George South wolves in comparison to (**A**) distance to linear features (m) and (**B**) linear feature density (km/km^2^) for summer (April 1–September 30) and winter (October 1–March 31), 2018–2020.

In both seasons, wolves had faster displacement rates when closer to linear features (Fig. 4A). However, wolf step length decreased as the density of linear features increased (Fig. 4B).

### Relationship between salvage logging features and moose kill-sites

**Figure 5:**
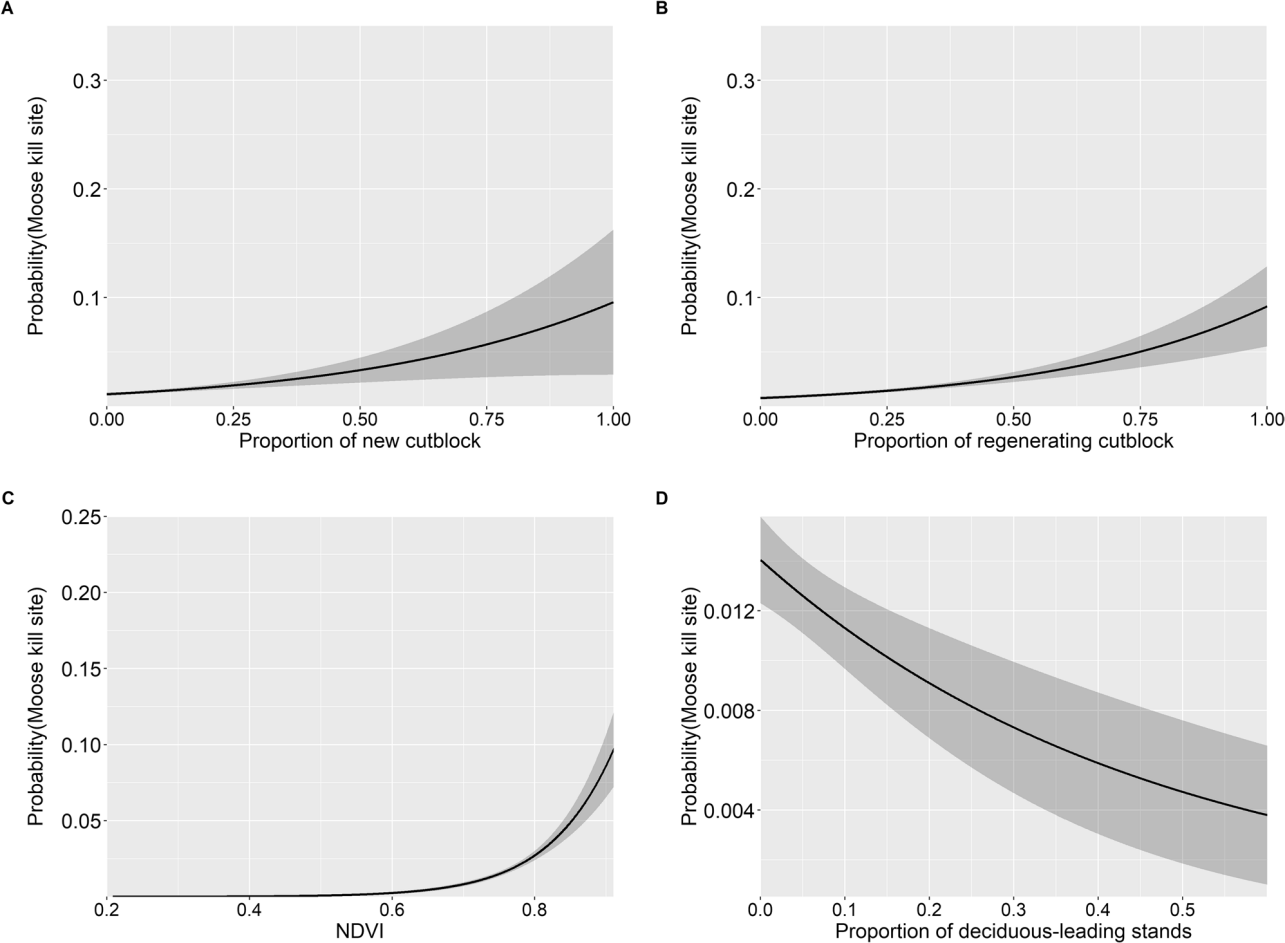
Predicted probability with 95% confidence intervals of a wolf kill-site of a moose occurring based on (**A**) proportion of new (0–8 years old) cutblocks, (**B**) proportion of regenerating (9–24 years old) cutblocks, and (**C**) mean normalized difference vegetation index (NDVI), and (**D**) proportion of deciduous-leading stands within a 883 m buffer around the location, in Prince George South, 2018–2020.

We identified 158 moose kill-sites using cluster analysis of wolf GPS locations (Supplementary Information S1). A single top model was best supported: “Prey + Cutblocks” (Table S4; r_s_ = 0.953). Moose kill-sites were more likely to occur in areas with higher proportions of new and regenerating cutblocks (Fig. 5A,B; Table 3). As mean NDVI increased, the probability of a moose kill-site occurring increased (Fig. 5C; Table 3). Moose kill-sites had a lower probability of occurring in areas with a higher proportion of deciduous-leading stands (Fig. 5D) and further from waterbodies (Table 3).

**Table 3:** Beta coefficient estimates for the top logistic regression model comparing habitat features to wolf kill-sites of moose in Prince George South, 2018–2020.

Covariate	Beta estimate	Standard error	Z value
Intercept	− 13.06	1.42	− 9.19
Pine	− 0.28	0.92	− 0.30
**Deciduous**	− 2.20	1.28	− 1.71
Mixed forest	− 0.46	0.67	− 0.69
Coniferous	− 4.69	4.77	− 0.98
**ln(Water)**	− 0.16	0.14	− 1.04
ln(Edge in)	0.065	0.096	0.49
ln(Edge out)	− 0.031	0.067	− 0.47
**NDVI**	12.17	1.86	6.53
**New cut**	2.25	0.85	2.66
**Regenerating cut**	2.60	0.58	4.44

Bolded terms indicate significance. ln() = log-transformed covariate.

## Discussion

Large-scale logging affects predator–prey dynamics by modifying predator search efficiency, elevating predation risk for prey near disturbance features. In our study, landscape change—cutblocks and linear feature networks—impacted wolf habitat selection and movement, altering the distribution of predation events to cutblocks. Based on our results, we suggest that extensive logging potentially creates an ecological trap mediated by season and cutblock age based on patterns of moose habitat selection identified in other studies within interior BC^14,33,34^. While this hypothesis requires further investigation, this scenario could contribute to moose population declines observed within the study area.

Wolf selection of new cutblocks, combined with an increased likelihood of moose kill-sites in areas with higher proportions of new and regenerating cutblocks, indicates that cutblocks are a risky feature for moose. In both seasons, wolves selected new cutblocks, suggesting better predation opportunities either due to higher prey availability or visibility^53,54^. Forage biomass increases post-harvest due to more solar insolation and nutrients available to plants, subsequently attracting ungulates^12,15,55–57^. However, forage biomass and ungulate use peaks a decade post-harvest^12,55^, and while moose kill-sites were linked to higher proportions of regenerating cutblocks, there was no trend in wolf selection for regenerating blocks. Regenerating cutblocks attract moose for the increased forage biomass and cover^12,14,33^, but increased vegetative cover would reduce prey visibility. Possibly, wolves are balancing prey availability and visibility in their selection of cutblocks, which is supported by our results: wolf movement rates were lower in regenerating cutblocks in summer, when wolf sightlines would be most obscured by vegetation. While wolves hunt with both olfactory and visual cues, areas with reduced cover (i.e. new cutblocks) are more likely to lead to a wolf successfully killing a moose due to both prey visibility and availability^58,59^, potentially leading to the observed selection of new cutblocks by wolves. However, adult female moose selection of new cutblocks—and thus, prey availability for wolves in these features—appears to vary based on season, with increased selection for new cutblocks in winter and avoidance in other seasons^14,33,34^. Consequently, moose vulnerability in new cutblocks is likely highest in winter due to their selection of these features, in addition to the presence of deeper snow. Wolf selection for new cutblocks throughout the year could indicate increased foraging success despite reduced moose availability in some seasons, which is supported by our kill-site analysis results. To clarify this, further studies could compare seasonal and demographic effects on spatial occurrence of wolf predation events of moose, which we were unable to do here due to limited sample sizes and data.

Consistent with previous research^1,5,27–29^, wolves selected for habitat near linear features and increased their displacement rates there. Linear features likely increase predation risk across the landscape by allowing predators to increase their search efficiency by facilitating movement^1,5,31^. Animals are predicted to spend less time in a foraging patch if the travel time between patches is reduced^60^ and therefore, linear features could promote faster searching of more habitat patches. Further, linear features provide travel corridors into refugia or biologically important habitat for ungulates, increasing spatial overlap between prey and predators^27,29^. As a result, predation risk may increase and homogenize across the landscape due to linear features.

We suggest that wolves use linear features as travel corridors into moose habitat which could enhance their chance of successfully detecting moose; however, linear features were not an important predictor of moose kill-sites. Unless used as human-created refugia^61^, ungulates generally avoid linear features due to perceived predation risk or limited forage availability relative to other habitats^34,50,62^. The combination of wolf selection for and moose avoidance of linear features^5^ likely interacts such that kill-sites are not necessarily close to linear features. Mumma and Gillingham^63^ also found that adult female moose were more likely to be killed by wolves in areas of low linear feature density. Therefore, kill-sites are not a function of linear features alone and our results suggest the cumulative effects of linear features and polygonal early-seral features produce the effect on kill-sites.

Despite selection for linear features, we observed no significant trend in wolf selection for areas of high linear feature densities. Previous research has identified inconsistent responses of wolves to varying densities of linear features^64–67^, which could be attributed to levels of human use—data which we lacked for PGS. While linear features may increase hunting efficiency of wolves, high linear feature densities are indicative of urban areas and increased accessibility of the landscape for human activities. If perceived as risky, areas with increased human activity would be avoided by wolves^65,68^. Alternatively, we were unable to differentiate between varying linear feature conditions (e.g., degree of vegetation growth) in the analysis and therefore, it is possible that this lack of trend in selection is an artefact of the dataset.

We propose that the behavioural responses to logging features by wolves coupled with cutblock forage attracting moose create conditions synonymous with an ecological trap for moose, mediated by season and cutblock age, although more research is required to conclude that a trap exists. Salvage logging creates a landscape with patches of attractive foraging habitats for ungulates (cutblocks), connected by a network of linear features that enable predator movement through the system, facilitating predation. Ungulates are attracted to the increased forage offered by regenerating vegetation in cutblocks^12,55–57^, but are more vulnerable to predation due to reduced cover^58^ and the ease of movement of predators through the system due to linear features^1,5^. Linear features increase spatial overlap of wolves and their prey by increasing accessibility of previously isolated habitat patches^27,29^ and allow wolves to efficiently search more of the landscape for prey^1,5^. If this potential ecological trap exists, it is likely mediated by season and cutblock age due to patterns in habitat selection by moose (i.e., increased selection for regenerating cutblocks; avoidance of new cutblocks except in winter; increased selection for cutblocks in winter) identified by previous moose research within interior BC^14,33,34^. Further investigation is required to characterize this potential ecological trap, by further assessing habitat preference and appropriate fitness and demographic measures for moose^69^.

Linear features and cutblocks function together to increase predation risk for prey, and effective management should target decoupling these disturbance features to reduce predator search efficiency. This could be accomplished by restoring linear features (e.g., felling trees, planting vegetation of > 1 m height^70,71^) that link cutblocks, to reduce wolf movement rates and access into moose habitat. Linear features linking biologically important but disjunct patches of moose habitat should be prioritized and if possible, construction of linear features should proactively avoid linking critical prey habitats. Habitat enhancement (e.g., planting palatable vegetation) should occur in areas where linear feature access is limited. Deciduous-leading stands may act as refuges for moose due to reduced wolf selection and fewer associated kill sites, and replanting or retention of these stands should be prioritized. Maintaining adequate cover for prey is important, by manipulating cutblock configuration to limit sightlines and decrease distance to cover, maintaining patches of intact forest (even dead standing pine), and allowing fast-growing shrubs to establish. However, shrub establishment may be a double-edged sword: while shrubs would disrupt predator sightlines and provide browse, they would encourage moose to use new cutblocks and potentially increase wolf-moose encounters. Overall, we emphasize the need to cohesively consider restoration and management of cutblocks and linear features in order to implement successful management programs, particularly in highly disturbed landscapes.

## Supplementary Information

Supplementary Information.

## Data Availability

Data are owned by the Government of British Columbia and are not publicly available at this time. Data requests can be directed to the Ministry of Environment and Climate Change Strategy Wildlife Species Inventory Team (SPI_Mail@gov.bc.ca). All spatial data is publicly available.
