# Supplementary material for: Cumulative effects of widespread landscape change alter predator–prey dynamics
Source: Sci Rep. 2022 Jul 8;12:11692. doi: 10.1038/s41598-022-15001-3 (PMC9270463; doi:10.1038/s41598-022-15001-3)
Supplement: Supplementary file 1 — Supplementary Information. [file 41598_2022_15001_MOESM1_ESM.docx]

# Supplementary Information

## Title: Cumulative effects of widespread landscape change alter predator-prey dynamics

Nicole P. Boucher^1*^, Morgan Anderson^2^, Andrew Ladle^1^, Chris Procter^3^, Shelley Marshall^4^, Gerald Kuzyk^5^, Brian M. Starzomski^1^, Jason T. Fisher^1^

^1^School of Environmental Studies, University of Victoria, Victoria, BC, V8W 2Y2 Canada

^2^ British Columbia Ministry of Forests, 2000 South Ospika Boulevard, Prince George, BC, V2N 4W5

^3^British Columbia Ministry of Forests, 1259 Dalhousie Drive, Kamloops, BC, V2C 5Z5

^4^British Columbia Ministry of Forests, 2080 Labieux Road, Nanaimo, BC, V9T 6J9

^5^Government of Saskatchewan Fish, Wildlife and Lands Branch, Ministry of Environment, Box 607, Unit #1 – 101 Railway Place, Meadow Lake, SK, Canada S9X 1Y5

*Corresponding author: nicoleboucher@uvic.ca, ORCID: [orcid.org/0000-0003-4125-654X](https://orcid.org/0000-0003-4125-654X)

## Study Area

The area is composed primarily of lodgepole pine (*Pinus contorta latifolia*) and hybrid spruce (*Picea glauca x engelmanni*) in the Sub-Boreal Spruce biogeoclimatic (BEC) zone, with the highest elevations characterized by the Engelmann Spruce Subalpine Fir BEC zone ^1^. Winter moose density declined in PGS from 630 ± 102/1000 km^2^ in 2011/2012 to 400 ± 78/100 km^2^ in 2016/2017 ^1^. Other ungulate species in the study area are mule deer (*Odocoileus hemionus*), white-tailed deer (*O. virginianus*), elk (*Cervus canadensis*), and domestic cattle (*Bos taurus*).

## Wolf Capture

All wolf captures were completed following Canadian Council on Animal Care guidelines and the British Columbia Wildlife Act (permit: PG17-272811). Immobilizations were completed using tiletamine-zolazepam (Telazol/Zoletil) at a 225 mg/mL concentration, with a volume of 1.5-1.8 mL administered at a 6 mg/kg dosage. For aerial darting, we used Pneudart 2-cc aerial darts with a 0.5” barb, injected into the wolf’s hindquarters. For net-gunning, wolves were captured using a net deployed from a hand-held net gun, then darted at a low power setting once the net restricted movement. Aerial darting and net-gunning were completed from Bell 206 helicopters. Trapping was completed on nights with temperatures >-8°C using steel foot-hold traps with rubberized offset jaws (Livestock Protection EZ Grip #7), with a drag hook on 8’ chain. Traps were placed on trails in areas with low risk to humans and domestic animals, with warning signs posted for the public. Attractants were used at the traps (gland lure, beaver castor, wolf urine and fresh wolf scats). Traps were checked in the morning or every 4-5 hours if ambient temperatures exceed 25°C (or traps were closed/removed). When trapped, wolves were restrained using a 4-6’ noose pole and chemically immobilized via hand-injection. All captured wolves were examined for previous or capture-related injury, blindfolded, and monitored for vital signs throughout handling.

## Integrated Step Selection Analysis

Step lengths were drawn from a gamma distribution parameterised using the step length data at the population level for each season, described by the tentative shape (influences the form/shape of the distribution; b_1_) and scale (influences amount of stretch or compression of gamma distribution; b_2_) parameters. Turn angles were sampled from a von Mises distribution (equal probability of left and right turns, with mean around zero). Integrated step selection analysis (iSSA) expands beyond previous step selection approaches by incorporating the log of the step length, ln(Step length), as a term within the model alongside habitat covariates, augmenting the tentative movement kernel which is no longer confounded by the process of habitat selection ^2^. Additionally, ln(Step Length) can be included as an interaction term to quantify the relationship between movement and habitat features.

Cutblock sizes and ages were determined using Reporting Silviculture Updates and Land Status Tracking System (RESULTS), obtained from DataBC (https://data.gov.bc.ca/). Cutblock ages were classified as new (0-8 years since harvest) and regenerating (9-24 years since harvest), based on average cutblock age thresholds identified by Mumma and Gillingham ^3^. In addition, we included the variables ‘edge in’ (distance to edge of forest when inside the forest) and ‘edge out’ (distance to edge of forest when outside of the forest) to describe use of edge habitat ^4,5^. We log-transformed both edge habitat variables. Forestry increases the amount of edge habitat within a landscape, and edge habitats may attract ungulates – and thus, wolves – due to their juxtaposition of hiding cover and foraging areas ^6,7^. One caveat is that these edge habitat variables contain limited information on how edge use varies based on edge boundary size. It is possible that wide edge boundaries would provide ecologically different features for wolves and their prey, as compared to narrow boundaries.

We used the Digital Road Atlas (DRA), obtained from DataBC (https://data.gov.bc.ca/), to calculate Euclidean distance (m) to the nearest linear feature from the start and end of the step. We log-transformed distance to the nearest linear feature. In addition, we created a raster of linear feature densities (km/km^2^) across the landscape and used this layer to extract linear feature density at the start and end of the step. Linear features attract wolves, particularly in the summer, due to facilitation of travel ^8,9^. Areas with high linear feature densities may be avoided by wolves due to increased human use ^10^. A caveat of this dataset is that it does not differentiate between the conditions of linear features (e.g. degree of vegetation growth).

Land cover was determined from the Vegetation Resources Inventory (VRI), obtained from the Ministry of Forests, Lands, Natural Resource Operations & Rural Development (FLNRORD) ^11^. Land cover was reclassified into categories of deciduous-leading stands, coniferous-leading stands, mixed forest stands, pine*-*leading stands (i.e., predominantly *Pinus* sp.), and non-forest (reference category). Deciduous-leading stands may be selected by wolves for hunting, as these stands provide forage for prey species, including moose in all seasons ^10^. Coniferous-leading stands provide shelter and snow interception for both wolves and their prey, as well as moose forage in the winter ^12,13^. Pine-leading stands were considered separate from other coniferous stands (spruce, fir), as we expected most pine to be impacted by MPB within this region. MPB-killed pine stands provide reduced shelter and snow interception, as compared to other coniferous-leading stands. We used the Water and Wetland GeoBase Land Cover dataset, obtained from DataBC (https://data.gov.bc.ca/), to determine the log-transformed distance to the nearest waterbody. Waterbodies can act as a travel corridor for wolves and riparian areas can provide hunting opportunities for beaver (*Castor canadensis*) and moose ^14^. Lastly, we used the R package ‘MODIStsp’ ^15^ to obtain MODIS (moderate resolution imaging spectroradiometer) 16-day interval normalized difference vegetation index (NDVI) data to represent plant productivity. NDVI values range between -1 to 1, with -1 representing water, 0 representing barren ground or rock, and values nearing 1 representing high plant productivity ^16^. In the summer, areas with high NDVI values may attract ungulates for foraging ^17^ and therefore, may be selected by wolves due to increased hunting opportunities. In winter, higher NDVI values represent vegetation with reduced snow cover (e.g. conifers) which could be selected by wolves for ease of travel, shelter, and hunting opportunities, while lower NDVI values represent areas with little vegetation or bare soil ^16^. We resampled all land cover layers to the coarsest resolution (250 m).

## Resource Selection Strength

We calculated resource selection strength using equations provided by Avgar, et al. ^18^. We calculated selection-free movement rates (km/hr) using the following formula:

$$\frac{1}{b_{2}}\times\left( b_{1}+\beta_{ln(Step length)}+{(\beta}_{1\ldots n}\times x_{1\ldots n}) \right)$$

Where b_1_ is the gamma shape parameter, b_2_ is the gamma scale parameter, ꞵ_ln(Step length)_ is the beta coefficient estimate for ln(Step length) and x_(1…_*_n_*_)_ is the beta coefficient estimates for interactions of ln(Step length) and covariates within the model.

## Moose mortality sites

We used the Find Points Cluster Identification Program (Version 2) ^19^ to identify potential kill sites for each wolf. Location clusters were determined using a 100 m search radius over 2 weeks (336 hours). The cluster algorithm was run monthly to identify possible kill sites.

Cluster sites with 15 wolf GPS locations or more were checked for signs of a kill site. Sites with smaller clusters were visited randomly. Moose killed between November and March were often buried by snow by the time the cluster algorithm was run so were typically visited following snow melt. Cluster sites were accessed using a truck or helicopter, but accessibility limited some site visits. As well, the COVID-19 pandemic limited or delayed field visits, particularly during 2020. Therefore, field visits to potential kill sites occurred days to months following clustering occurrence or were not always possible.

At each potential kill site, the area was searched for evidence of prey species (bones, hair), sex (antlers or pedicels, pelvis) and age (size, tooth eruption and wear, incisor for cementum annuli aging). Searches started at the cluster centroid and occurred within a 100-m radius, with a focus on landscape features (e.g., game trails, wolf bed sites) where evidence of a kill may be left.

The identification of carnivore kill sites of large-bodied prey species like moose using GPS cluster analysis is a common and reliable method; however, several assumptions exist in identifying kill sites using this method ^20-23^. We assumed that if a wolf killed a moose, handling time of the carcass would be enough for the kill site to be detected as a cluster. However, GPS cluster analysis may not identify sites with quickly consumed kills or where wolves are displaced from a kill. We assumed that evidence of a kill would be present at each kill site; however, it is possible that false negatives occurred due to carcasses being fully consumed, minimal remains being undetected under cover or carcasses having been moved by scavengers. These challenges are magnified for small-bodied prey like moose neonates, and a 1-hour fix schedule is unlikely to detect these kill sites ^21,23-25^. As such, we are not able to assess the landscape characteristics that are more likely to lead to wolf predation on neonates. Lastly, while we identified and removed scavenged bear kills and hunter kills from the data set, we assumed that all remaining kill sites were wolf kills. However, it is possible that some of these moose were scavenged.

A total of 4330 potential kill sites were identified using GPS cluster analysis. Of these, we were able to search 292 of 497 sites of clusters with ≥15 wolf locations and 305 of 3833 sites with <15 wolf GPS points. In total, 597 cluster locations were searched. Clusters were investigated on average 99.3 ± 2.9 days (mean ± SE) following the latest date the kill could have occurred.

From the ground-truthed clusters, we identified 158 kill sites of moose. Of these, 145 kill sites of moose were identified from clusters with ≥15 wolf GPS locations. As well, we identified kill sites of other species (black bear = 2, cattle = 3, deer = 4, elk = 1), 18 rendezvous sites, 8 den sites, 39 active beaver areas, 3 bait sites, and 1 moose-wolf stand-off site.

On average, kill sites of moose were 4.16 ± 0.37 m (mean ± SE) from the centroid of the wolf GPS location cluster. Kill sites were primarily of adult moose (n = 112), followed by calves >3 months old (n = 38), and 8 moose of an unknown age class. The sex of most carcasses was unknown (n = 80), but 61 were identified as female.

We generated fifty random locations ^26^ for each mortality site within the PGS study area boundary, and buffered each location by 883 m – the average successful pursuit distance of a moose by a wolf ^27^. Within each buffer, we determined the proportion of deciduous-leading stands, coniferous-leading stands, mixed forest stands, pine*-*leading stands, new cutblocks, and regenerating cutblocks. We determined mean values for distance to linear feature, linear feature density, distance to edge (both inside and outside of the forest), NDVI and distance to water. We log-transformed all distance variables. Due to the limited sample size, we pooled kill sites from all wolf individuals for analysis.

Table S1. Summary of radio-collared wolves with movement data for summer (April 1 – September 30) and winter (October 1 – March 31), 2018-2020. Wolves were only included within the analysis if sufficient data (>7 collared days) was available.

| ID | Pack | Sex | Winter 2018 | Summer 2018 | Winter 2019 | Summer 2019 | Winter 2020 | Summer 2020 | Fate |
| --- | --- | --- | --- | --- | --- | --- | --- | --- | --- |
| 1 | A | M | x |  |  |  |  |  | Dispersed |
| 2 | B | M |  | x | x |  |  |  | Dispersed/shot |
| 3 | C | M | x | x | x |  |  |  | Offline |
| 4 | D | M | x | x | x |  |  |  | Dispersed |
| 5 | E | M |  |  | x | x | x |  | Offline |
| 6 | D | M |  |  | x | x |  |  | Dispersed |
| 7 | E | M |  |  |  | x | x | x | Active |
| 8 | D | F |  |  |  |  |  |  | Shot; insufficient data |
| 9 | F | M |  |  |  |  | x | x | Offline |
| 10 | G | M |  |  |  |  | x | x | Active |
| 11 | F | F |  |  |  |  |  | x | Offline |

Table S2. Candidate models examining the relationship between salvage logging features and successful wolf kills of moose in the Prince George South study area, 2018-2020.

| Model name | Covariates |
| --- | --- |
| Prey | Pine + Deciduous + Mixed Forest + Coniferous + ln(Distance to water) + NDVI + ln(Edge in) + ln(Edge out) |
| Linear feature network | Linear feature density + ln(Distance to linear feature) |
| Cutblock | New cut + Regenerating cut + New cut:Cut size + Regenerating cut:Cut size |
| Prey + Linear feature network | Pine + Deciduous + Mixed Forest + Coniferous + ln(Distance to water) + NDVI + ln(Edge in) + ln(Edge out) + Linear feature density + ln(Distance to linear feature) |
| Prey + Cutblock | Pine + Deciduous + Mixed Forest + Coniferous + ln(Distance to water) + NDVI + ln(Edge in) + ln(Edge out) + New cut + Regenerating cut + New cut:Cut size + Regenerating cut:Cut size + ln(Step length):New cut |
| LFN + Cutblock | Linear feature density + ln(Distance to linear feature) + New cut + Regenerating cut + New cut:Cut size + Regenerating cut:Cut size |
| Global | Pine + Deciduous + Mixed Forest + Coniferous + ln(Distance to water) + NDVI + ln(Edge in) + ln(Edge out) + Linear feature density + ln(Distance to linear feature) + New cut + Regenerating cut + New cut:Cut size + Regenerating cut:Cut size |

Table S3. Spearman’s rank correlation coefficient (r_s_) medians from cross validations assessing fits of integrated step selection analysis models for each wolf individual in summer (April 1 – September 30) and winter (October 1 – March 31) in Prince George, 2018-2020.

| Season | Model | r_s_ |
| --- | --- | --- |
| Summer | Prey | 0.74 |
|  | Linear feature network | 0.72 |
|  | Cutblock | 0.61 |
|  | Prey + Linear feature network | 0.77 |
|  | Prey + Cutblock | 0.76 |
|  | LFN + Cutblock | 0.76 |
|  | Global | 0.81 |
| Winter | Prey | 0.57 |
|  | Linear feature network | 0.48 |
|  | Cutblock | 0.35 |
|  | Prey + Linear feature network | 0.57 |
|  | Prey + Cutblock | 0.58 |
|  | LFN + Cutblock | 0.56 |
|  | Global | 0.66 |

Table S4. Model selection using Akaike’s information criterion (AIC) and for logistic regressions relating habitat features to wolf kill-sites of moose in Prince George South, 2018-2020. LFN = linear feature network.

| Model | K | AIC | ΔAIC | AIC Weight | Log likelihood |
| --- | --- | --- | --- | --- | --- |
| Prey + Cutblock | 11 | 1318.21 | 0 | 0.87 | -648.09 |
| Global | 13 | 1322.03 | 3.82 | 0.13 | -647.99 |
| Prey | 9 | 1336.12 | 17.91 | 0 | -659.05 |
| Prey + LFN | 11 | 1338.85 | 20.64 | 0 | -658.41 |
| LFN + Cutblock | 5 | 1362.51 | 44.31 | 0 | -676.25 |
| Cutblock | 3 | 1364.29 | 46.08 | 0 | -679.14 |
| LFN | 3 | 1394.28 | 76.07 | 0 | -694.14 |


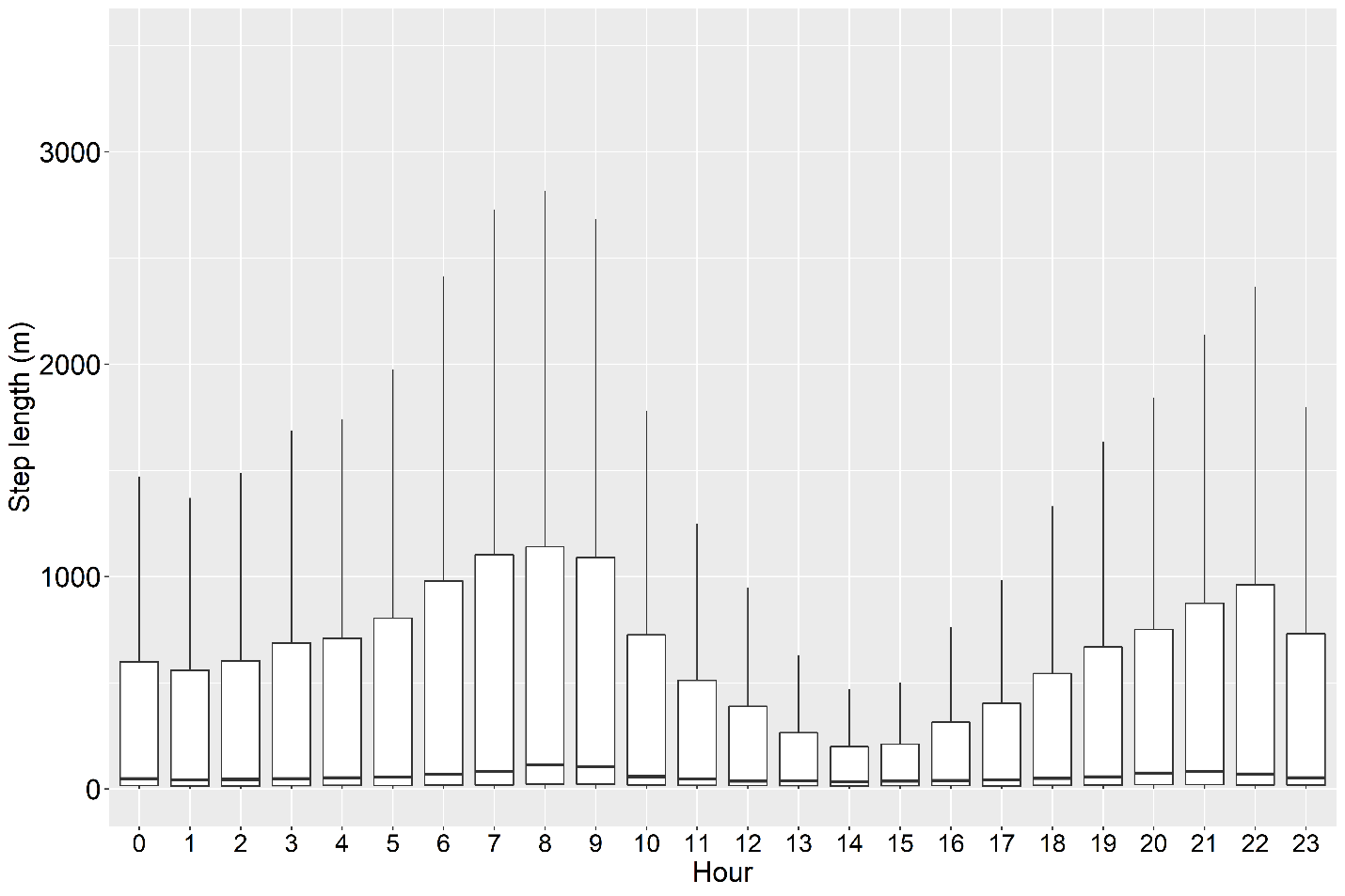


Figure S1. Variation in step length (m) by hour for wolves in Prince George South, 2018-2020.

## References

1 Procter, C. *et al.* Factors affecting moose population declines in British Columbia. 2020 Progress Report: February 2012-May 2020. B.C. Ministry of Forests, Lands, Natural Resource Operations and Rural Development, Victoria, B.C., Wildlife Working Report No. WR-128. Pp. 89. <https://www2.gov.bc.ca/gov/content/environment/plants-animals-ecosystems/wildlife/wildlife-conservation/moose/moose-conservation/moose-research>. (2020).

2 Avgar, T., Potts, J. R., Lewis, M. A. & Boyce, M. S. Integrated step selection analysis: bridging the gap between resource selection and animal movement. *Methods in Ecology and Evolution* **7**, 619-630, doi:10.1111/2041-210X.12528 (2016).

3 Mumma, M. & Gillingham, M. Determining factors that affect survival of moose in Central British Columbia. Technical report to the Habitat Conservation Trust Foundation for Grant Agreement CAT19-0-522 (1 April 2017 through 31 March 2019). 56 (2019).

4 Ladle, A. *et al.* Grizzly bear response to spatio-temporal variability in human recreational activity. *J. Appl. Ecol.* **56**, 375-386, doi:10.1111/1365-2664.13277 (2019).

5 Northrup, J. M. *et al.* Vehicle traffic shapes grizzly bear behaviour on a multiple‐use landscape. *J. Appl. Ecol.* **49**, 1159-1167 (2012).

6 Tinoco Torres, R., Carvalho, J. C., Panzacchi, M., Linnell, J. D. C. & Fonseca, C. Comparative use of forest habitats by roe deer and moose in a human-modified landscape in southeastern Norway during winter. *Ecol. Res.* **26**, 781-789, doi:10.1007/s11284-011-0837-0 (2011).

7 Mysterud, A. & Østbye, E. Cover as a habitat element for temperate ungulates: effects on habitat selection and demography. *Wildlife Society Bulletin (1973-2006)* **27**, 385-394 (1999).

8 Dickie, M., McNay, S. R., Sutherland, G. D., Cody, M. & Avgar, T. Corridors or risk? Movement along, and use of, linear features varies predictably among large mammal predator and prey species. *J. Anim. Ecol.* **n/a**, doi:10.1111/1365-2656.13130 (2019).

9 Dickie, M., Serrouya, R., McNay, R. S. & Boutin, S. Faster and farther: wolf movement on linear features and implications for hunting behaviour. *J. Appl. Ecol.* **54**, 253-263 (2017).

10 Houle, M., Fortin, D., Dussault, C., Courtois, R. & Ouellet, J.-P. Cumulative effects of forestry on habitat use by gray wolf (*Canis lupus*) in the boreal forest. *Landsc. Ecol.* **25**, 419-433, doi:10.1007/s10980-009-9420-2 (2010).

11 Ministry of Forests, L., Natural Resource Operations and Rural Development. *Vegetation Resources Inventory*, <<https://catalogue.data.gov.bc.ca/dataset/vri-2019-forest-vegetation-composite-rank-1-layer-r1->> (2020).

12 Fortin, D. *et al.* Wolves influence elk movements: behavior shapes a trophic cascade in Yellowstone National Park. *Ecology* **86**, 1320-1330, doi:10.1890/04-0953 (2005).

13 Kittle, A. M., Fryxell, J. M., Desy, G. E. & Hamr, J. The scale-dependent impact of wolf predation risk on resource selection by three sympatric ungulates. *Oecologia* **157**, 163-175 (2008).

14 Newton, E. J. *et al.* Compensatory selection for roads over natural linear features by wolves in northern Ontario: Implications for caribou conservation. *PLOS ONE* **12**, e0186525, doi:10.1371/journal.pone.0186525 (2017).

15 Busetto, L. & Ranghetti, L. MODIStsp: An R package for automatic preprocessing of MODIS Land Products time series. *Computers & geosciences* **97**, 40-48 (2016).

16 Neigh, C. S., Tucker, C. J. & Townshend, J. R. North American vegetation dynamics observed with multi-resolution satellite data. *Remote Sens. Environ.* **112**, 1749-1772 (2008).

17 Pettorelli, N. *et al.* The Normalized Difference Vegetation Index (NDVI): unforeseen successes in animal ecology. *Clim. Res.* **46**, 15-27 (2011).

18 Avgar, T., Lele, S. R., Keim, J. L. & Boyce, M. S. Relative selection strength: quantifying effect size in habitat- and step-selection inference. *Ecology and Evolution* **7**, 5322-5330, doi:10.1002/ece3.3122 (2017).

19 Gillingham, M. Documentation for using Find Points Cluster Identiﬁcation Program (Version 2). University of Northern British Columbia, Prince George. (2009).

20 Anderson, C. R. & Lindzey, F. G. Estimating cougar predation rates from GPS location clusters. *J. Wildl. Manag.* **67**, 307-316, doi:10.2307/3802772 (2003).

21 Sand, H., Zimmermann, B., Wabakken, P., Andrèn, H. & Pedersen, H. C. Using GPS technology and GIS cluster analyses to estimate kill rates in wolf‐ungulate ecosystems. *Wildl. Soc. Bull.* **33**, 914-925 (2005).

22 Morehouse, A. T. & Boyce, M. S. From venison to beef: seasonal changes in wolf diet composition in a livestock grazing landscape. *Frontiers in Ecology and the Environment* **9**, 440-445 (2011).

23 Webb, N. F., Hebblewhite, M. & Merrill, E. H. Statistical methods for identifying wolf kill sites using Global Positioning System locations. *J. Wildl. Manag.* **72**, 798-807, doi:10.2193/2006-566 (2008).

24 Merrill, E. *et al.* Building a mechanistic understanding of predation with GPS-based movement data. *Philosophical transactions of the Royal Society of London. Series B, Biological sciences* **365**, 2279-2288, doi:10.1098/rstb.2010.0077 (2010).

25 Gable, T. D., Windels, S. K., Bruggink, J. G. & Homkes, A. T. Where and how wolves (Canis lupus) kill beavers (Castor canadensis). *PLoS One* **11**, e0165537 (2016).

26 Northrup, J. M., Hooten, M. B., Anderson Jr, C. R. & Wittemyer, G. Practical guidance on characterizing availability in resource selection functions under a use–availability design. *Ecology* **94**, 1456-1463 (2013).

27 Paquet, P. C. Behavioral ecology of sympatric wolves (Canis lupus) and coyotes (C. latrans) in Riding Mountain National Park, Manitoba. (1990).
